# Self-regulation training generalizability using the regulation of craving task. An fMRI study

**DOI:** 10.3389/fpsyg.2024.1399456

**Published:** 2024-07-23

**Authors:** Iriannys Torres Morillo, Marcia Smith Pasqualini, Morgan G. Brucks, Laura E. Martin

**Affiliations:** ^1^Avila University, Kansas City, MO, United States; ^2^Hoglund Biomedical Imaging Center, University of Kansas Medical Center, Kansas City, KS, United States; ^3^Department of Population Health, University of Kansas Medical Center, Kansas City, KS, United States; ^4^Cofrin Logan Center for Addiction Research and Treatment, University of Kansas, Lawrence, KS, United States

**Keywords:** self-regulation, food craving, brain activation, addiction, fMRI

## Abstract

Individual differences in reward salience may relate to the difficulty in regulating the effects of multiple substances (e.g., nicotine and food). Increased brain activation in reward and self-regulation (SR) regions has been evidenced while adults view appetitive cues (e.g., food pictures) to test substance use disorder treatment response. Enhancing SR with behavioral interventions may increase brain activation in SR regions and reduce responses in reward regions. Our primary analysis demonstrated increased brain activation in SR regions to smoking cues among individuals who practiced SR by delaying their first cigarette of the day for 2 weeks. However, little is known about the generalizability of SR between appetitive cues. This secondary analysis explored the influence of adherence to a SR behavioral intervention by examining the impact of practicing smoking SR on brain activation to food cues among adults who smoke. Participants (*N* = 65) were randomly assigned to practice SR by delaying their first daily cigarette or smoking as usual for 2-weeks. Functional magnetic resonance imaging data were collected while people were told to think of “negative” or “positive” associations with the cue. The results indicated that practicing smoking SR was linked with increased activation in the dorsolateral prefrontal cortex (dlPFC) when viewing food cues. There was no correlation between delaying smoking adherence and brain activation in the dlPFC. Exploratory analyses suggested higher dlPFC activation when people thought about “positive” associations with the food cues instead of “negative” ones. We concluded that practicing smoking SR is related to increased brain activation to food cues, suggesting potential generalizability of SR practice from smoking cues to food cues.

## 1 Introduction

Reward processing and self-regulation (SR) brain regions show increased activation in responses to appetitive cues, such as pictures of food or pictures of cigarettes for individuals who smoke. Behavioral interventions may strengthen SR, thus increasing SR brain responses to appetitive cues. Our primary study examined the impact of practicing SR around smoking on brain responses to smoking cues (Fox et al., 2018) and found increases in SR related brain activation to smoking cues. However, little is known regarding the generalizability of SR from one appetitive cue (e.g., food) to another (e.g., cigarettes).

Self-regulation (SR) is defined as the ability to control responses to the surrounding environment. The dual systems model of SR (Cohen, 2017) delineates the interaction between two independent cognitive systems. System I is the automatic system that responds to temptations and System II is the deliberative system that helps assign appropriate behavioral responses. Other models of SR consider the multiple intrinsic and extrinsic factors that play a role in goal achievement (Inzlicht et al., 2021). Neal et al. (2017) discussed a dynamic view of SR, where individuals juggle multiple-goal pursuits simultaneously while making corresponding decisions to prioritize each, following their realistic values, time constraints, and personal self-efficacy. Although SR is limited, people can divide their attention span to achieve goals, resulting in split levels of SR for each objective. In contrast, the ego-depletion model by Muraven and Baumeister (2000) compares SR to muscle fatigue: it is viewed as an ability that has the potential to strengthen with practice. This implies that SR is limited and only becomes “stronger” if time and mental effort are dedicated to reinforcing it to a specific cue. In sum, all models are goal-oriented, but dual system and dynamic views explain SR processes as generalizable across cues, whereas ego-depletion defines them as cue-specific.

### 1.1 The use of cue-reactivity paradigms to study craving and self-regulation

Cue reactivity is a method to understand craving within experimental situations by presenting individuals with craving-inducing cues, such as cigarettes for someone who smokes (Sayette and Tiffany, 2013). Craving is a cognitively emotional event that drives desire for a substance (Kavanagh et al., 2013). Cue-reactivity paradigms can be used to assess an individual’s craving and urges by exposing them to cues and observing their psychological and physiological responses after exposure (Drummond, 2000). Based on behavioral learning theories and substance use research, contextual cues (e.g., social settings, body awareness) can activate arousal, making the brain re-create previous memories and reinforcing cue-related behavior (LeCocq et al., 2020). Thus, the stimulus is assigned incentive salience, which will motivate the individual to pay excessive attention to that stimulus in the near environment, possibly leading to substance use behaviors (Olney et al., 2018). When it comes to food cues, individuals also present physiological (e.g., salivation), psychological (e.g., craving) and neural responses (e.g., brain activation) as consequences of the learning process preceding food intake (Van den Akker et al., 2014). Using fMRI paradigms in cue-evoked-reactivity to study eating behaviors allows detection of neural mechanisms associated with food seeking/consumption behaviors. Cosme et al. (2023) found that the most robust methods to show associations between eating behaviors and body composition were cue reactivity and valuation. They used a cue reactivity task in an fMRI study to compare multiple indicators of SR, reward and craving reactivity, and valuation. Individuals were asked to indicate which images of appetizing food, nature, and social scenarios were held indoors or outdoors, but were not asked to regulate their food cravings. Neuroimaging studies of cue-reactivity can examine brain activation to cues (e.g., food) and brain regions involved in responding to cues and controlling reward-seeking behaviors while exploring substance use disorder treatment response in adults (Courtney et al., 2016). The neural reward systems respond to pleasurable stimuli that activate the dopaminergic pathway and reinforce the memory of the reward promoting repeated reward seeking (Berridge, 2007). The neural systems of SR including the dorsolateral prefrontal cortex (dlPFC) play a role in regulation, inhibitory control and goal-directed behaviors by managing automatic responses and redirecting attentional focus to give a controlled response (Friedman and Robbins, 2022). A methodological review by Ekhtiari et al. (2016) found that when assessing cognitive control, fMRI paradigms revealed a predominance of top–down cortical control by the prefrontal cortex, denoting it as the primary pathway for craving and emotional regulation over other brain regions. Cue-reactivity paradigms in fMRI allowed the assessment of drug craving in laboratory settings because they remove the obstacle of determining the explicit or implicit nature of the craving. A few studies (Eichhammer et al., 2003; Fregni et al., 2008) found that both positive and negative transcranial direct current stimulation (tDCS) on the dlPFC effectively reduced of fixation and consumption of food and drugs compared to using Sham stimulation. These findings suggest that the dlPFC could be involved in regulating reward from multiple cues.

### 1.2 Craving and reward systems

When assessing SR for food in individuals who smoke, we must consider how nicotine affects food craving. Nicotine possesses addictive properties and impacts hormones and neurotransmitters associated with reward and satiety processes, insulin regulation, internal bodily stress responses, and energy expenditure, thus influencing metabolism (Harris et al., 2016). It also decreases appetite, research suggests that some individuals continue smoking for weight management (Camp et al., 1993). People who smoke may face additional challenges when regulating their cravings towards appetitive cues. Studies among adults who smoke have shown that suppressing appetite and controlling overeating is a motive for smoking (Bloom et al., 2019). The Center for Disease Control and Prevention (2023) has found that some individuals going through withdrawal from nicotine during a quit attempt report feeling more tired, hungry, having difficulty focusing, experience mood changes and stronger cravings for tobacco and food (Komiyama et al., 2013; Harris et al., 2016). This suggests that individuals who are attempting to quit smoking may need to regulate their responses to both smoking and food cues due to nicotine withdrawal. Determining whether SR is cue-specific, or a generalizable skill could be beneficial, because it would shift the focus from what substance is consumed to how the SR training is applied.

## 2 Purpose of the present study

The goal of this study was to explore whether SR processes are generalizable. The parent CO Practice Experiment (COPE) study examined whether people who smoke could strengthen their self-regulation abilities towards smoking cues following daily practice regulating smoking urges by delaying the first cigarette of the day. The results demonstrated that individuals randomized to delay their first cigarette of the day showed greater change in brain activation in the dorsolateral prefrontal cortex to smoking cues compared to individuals who were randomized to smoke as usual. The findings suggest that self-regulation practice around smoking behaviors increases self-regulation related brain activation to smoking cues. In the current secondary analysis, we examined SR to food cues in individuals who smoke to see if practicing SR related to smoking cues generalized to SR for food cues. Increasing SR should help individuals develop a stronger sense of self-awareness, allowing them to identify body signals (e.g., craving) more accurately. They could then allocate SR resources to take steps to reach their goals of controlling or limiting cue-driven patterns of behaviors (e.g., smoking, eating). We hypothesized that among a sample of individuals who smoke, practicing SR related to smoking (i.e., delaying the first cigarette of the day) would lead to changes in SR related brain activation (i.e., dlPFC) when viewing food cues, suggesting generalization of SR from smoking cues to food cues. In addition, we hypothesized a positive correlation between adherence to SR practice (i.e., percentage of days where they successfully followed their smoking schedule) and brain activation changes in dlPFC activation while viewing food cues.

## 3 Method

### 3.1 Participants

The sample in the primary study consisted of 65 adults who were randomized to either practice SR or smoke as usual. Inclusion criteria included smoking at least 10 cigarettes per day, smoking within 90 min of waking, age 18–60 years, normal or corrected to normal vision, no plan to quit smoking during the previous or the next 30 days, and willingness to delay their first cigarette of the day each day, for at least 2 weeks. The exclusion criteria included pregnancy, 50 or higher body mass index (due to participant comfort in the MRI scanner), left-handedness, MRI contraindications, medical history of general, neurological, or mental illnesses (except for anxiety, depression, or attention deficit hyperactivity disorder), history of concussions, currently taking seizure medication, history of anorexia nervosa or bulimia nervosa. Follow-up MRI data were missing for the following reasons: lost to follow-up (*n* = 5); claustrophobia (*n* = 1); surgery between MRI appointments (*n* = 1), sleeping during the MRI (*n* = 6), and COVID-19 shutdown (*n* = 1). Five participants were lost to follow-up. The final dataset included 65 participants with 32 individuals in the practice group (*n* = 8 missing follow-up MRI) and 33 in the non-practice group (*n* = 5 missing follow-up MRI). The socio-demographic characteristics showed group ages ranged between 20 and 60 years old (*M* = 36.1, *SD* = 11.0). They were comprised of 50.8% female and 47.4% male. Of these, 78.5% identified as white, 13.8% as Black or African American, and 3.1% as native American; 4.6% did not answer. Lastly, 91% of the sample was ethnically classified as non-Hispanic.

### 3.2 Procedures

Participants were enrolled in a 4-week study. Following informed consent, demographics information and baseline questionnaires were completed. Participants were given a carbon monoxide (CO) monitor and instructed to use it upon waking, before their first cigarette, and after their first cigarette, for 1 week. These data were used to objectively identify the average time from waking to smoking the day’s first cigarette. CO data were recorded on a paper packet provided to the participant and saved on the CO monitor. Following 1 week of monitoring, CO participants returned their monitor and completed baseline measures of SR, craving, and the fMRI regulation of craving task described below. Following the fMRI, participants received tips on regulating craving and were randomly assigned to either practice regulation around smoking by delaying the first cigarette of the day for the next 2 weeks or assigned to smoke as usual for the next 2 weeks. Participants in the Practice group were asked to increasingly delay smoking their first cigarette of the day by up to 2 hours per day for 2 weeks. They were provided with a personalized smoking schedule based on smoking habits that progressively delayed the first cigarette of the day for 2 weeks. The Control group continued their regular smoking habits. Participants were compensated after each visit and were given an additional bonus after completing all study activities. Participants completed a second fMRI session following 2 weeks of delaying the first cigarette of the day or smoking as usual. During the 2-week period between fMRI sessions, participants continued to use the CO monitor upon waking, immediately before and after the first and second cigarette of the day. A full list of questionnaires and procedures can be found in Fox et al. (2018). All study procedures were approved by the University of Kansas Human Subjects Committee (STUDY00004095).

### 3.3 Measures

#### 3.3.1 Regulation of craving task

**Figure 1:**
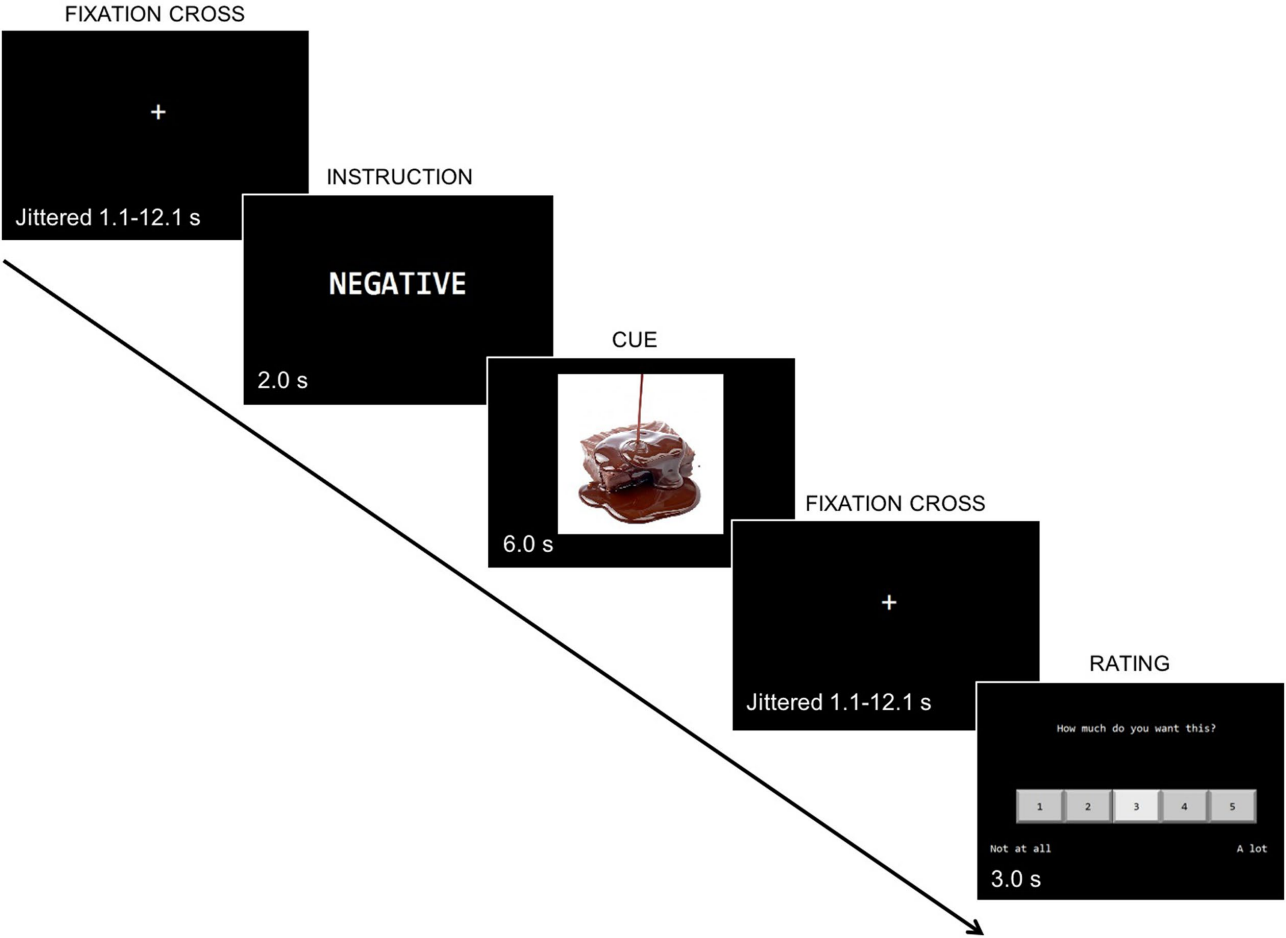
Regulation of craving task in the fMRI (food-negative version) variation of Kober et al. (2010) fMRI paradigm. First, participants were presented with a fixation cross, then an instruction indicating they had to think about positive or negative consequences of consuming a food item, followed by an appetitive cue, and another fixation cross. They then rated with a Likert scale how much they wanted to consume the item from (1 = “*Not at all*” to 5 = “*A lot”*).

On the Regulation of Craving Task (ROC) (see Figure 1), each trial consisted of a one-word instruction (“Positive” or “Negative”) followed by an appetitive cue (smoking or food). Participants were instructed to think about the “positive” or “negative” consequences of consuming the presented cue. Based on emotion regulation paradigms, thinking about the “negative” consequences should engage regulation related brain regions to a greater degree than thinking about “positive” consequences of consuming the item presented. Examples of both “positive” and “negative” conditions were provided to help them understand the task. Following the cue, participants were asked to rate with a 5-point Likert scale how much they desired to consume the item (1 = “*Not at all*” to 5 = “*A lot”*). Prior to entering the scanner, participants completed a practice session to become familiar with the task instructions and ask questions.

The ROC consisted of 5 fMRI runs lasting about 6 min each with breaks provided between runs. Twenty trials were presented during each run. The images were equally split between positive/negative consequences and food/smoking cues. Food images were selected from stock photo images and assessed for valence, arousal, and appetizing level (Szabo-Reed et al., 2015). The smoking images were selected from publicly available images on the internet as well as the International Smoking Image Series (Gilbert and Rabinovich, 2003), The International Affective Picture System (McClernon et al., 2005).

**Figure 2:**
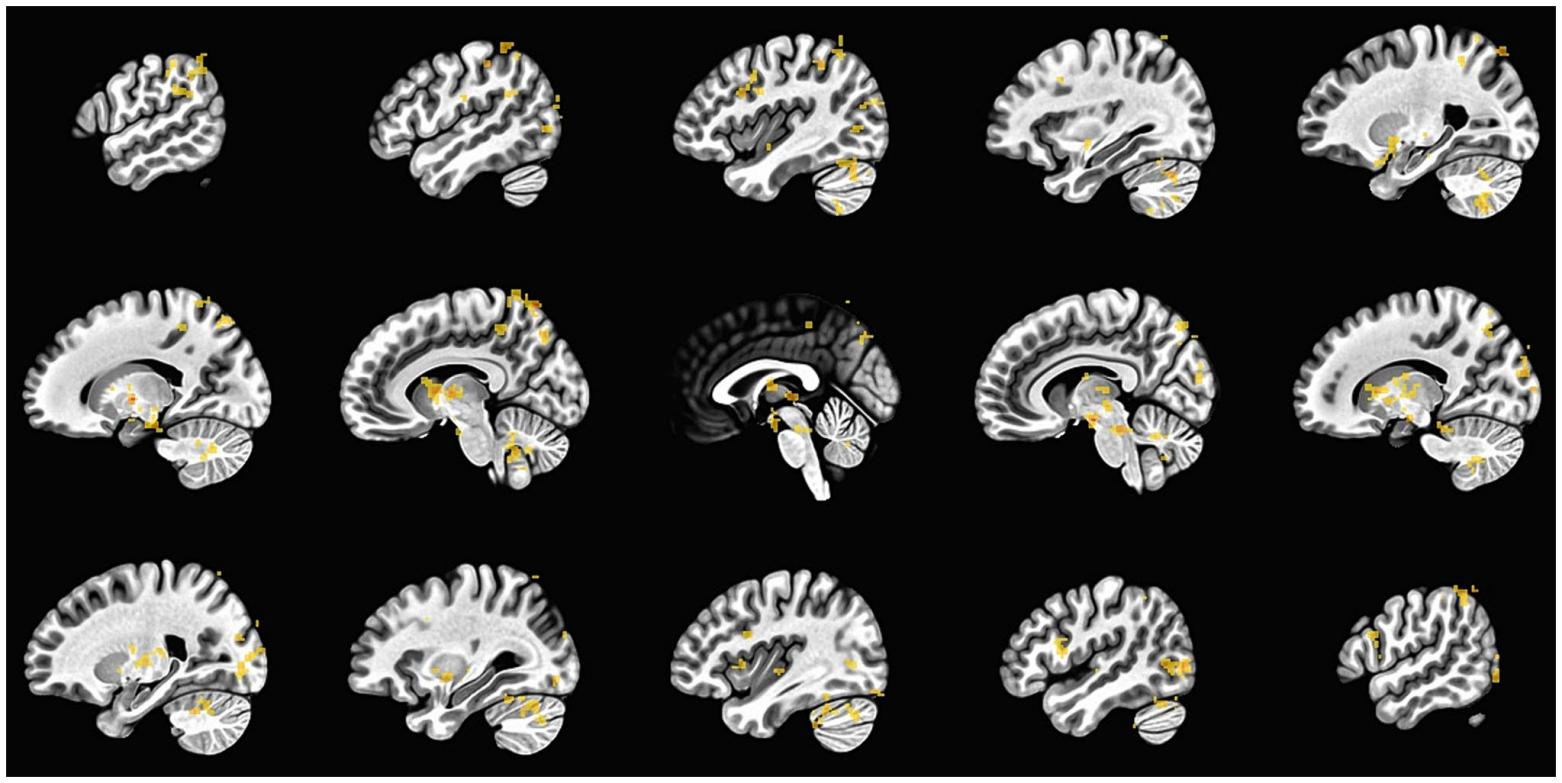
Whole brain analysis in response to food-cue reactivity.

After the fMRI, participants reported to the research staff what they were thinking about when deciding how much they wanted to consume such items. Participants reported thinking about texture, smell, taste, health factors, personal preferences and how that food made them feel. In addition, the “positive” instruction included reports regarding temperature and social–emotional factors. For the negative instruction, different categories emerged, such as body image, feelings regarding their dietary progress, economic and environmental impact. An exploratory whole brain analysis was performed to show activation in response to food-cue reactivity during the ROC task Figure 2.

#### 3.3.2 Functional magnetic resonance imaging (fMRI) acquisition

Scanning was performed on a 3-tesla Siemens Skyra scanner (Siemens, Erlangen, Germany) fitted with a 20-channel head and neck coil. Following automated scout image acquisition and shimming procedures to optimize field homogeneity, resting state, fMRI task, and a structural scan were acquired. The current analysis focuses only on the ROC Task. Resting state scanning parameters included BOLD sequences of 52 contiguous slices at a 40° angle to the anterior commissure–posterior commissure (AC-PC) line (repetition time/echo time [time repetition (TR)/time echo (TE)] = 3000/25 ms, flip angle = 90°, field of view = 640 × 640, matrix = 80 × 80, slice thickness = 3 mm, in plane resolution = 2.9 mm, 200 data points). ROC Task scanning parameters included gradient echo BOLD scans (five, one for each run of the craving rating task), which were acquired in 43 contiguous oblique axial slices at a 40° angle (TR/TE = 2500/25 ms, flip angle = 90°, field of view = 560 × 560 mm, matrix = 80 × 80, slice thickness = 3.0 mm, in-plane resolution = 2.9 × 2.9 mm, 145 data points). Finally, a T1-weighted structural scan was completed (3D MPRAGE sequence, TR/TE = 2300/2.95 ms, flip angle = 9°, field of view = 253 × 270 mm, matrix = 240 × 256, slice thickness = 1.2 mm, 176 slices). This scan was used for spatial normalization and coregistration with fMRI data. All participants were positioned in the scanner so that the angle of the anterior to posterior commissure plane was between 17° and 22° to the scanner coordinate space, ensuring that the 40° lice acquisition angle was constant for all participants.

#### 3.3.3 Statistical analysis

**Figure 3:**
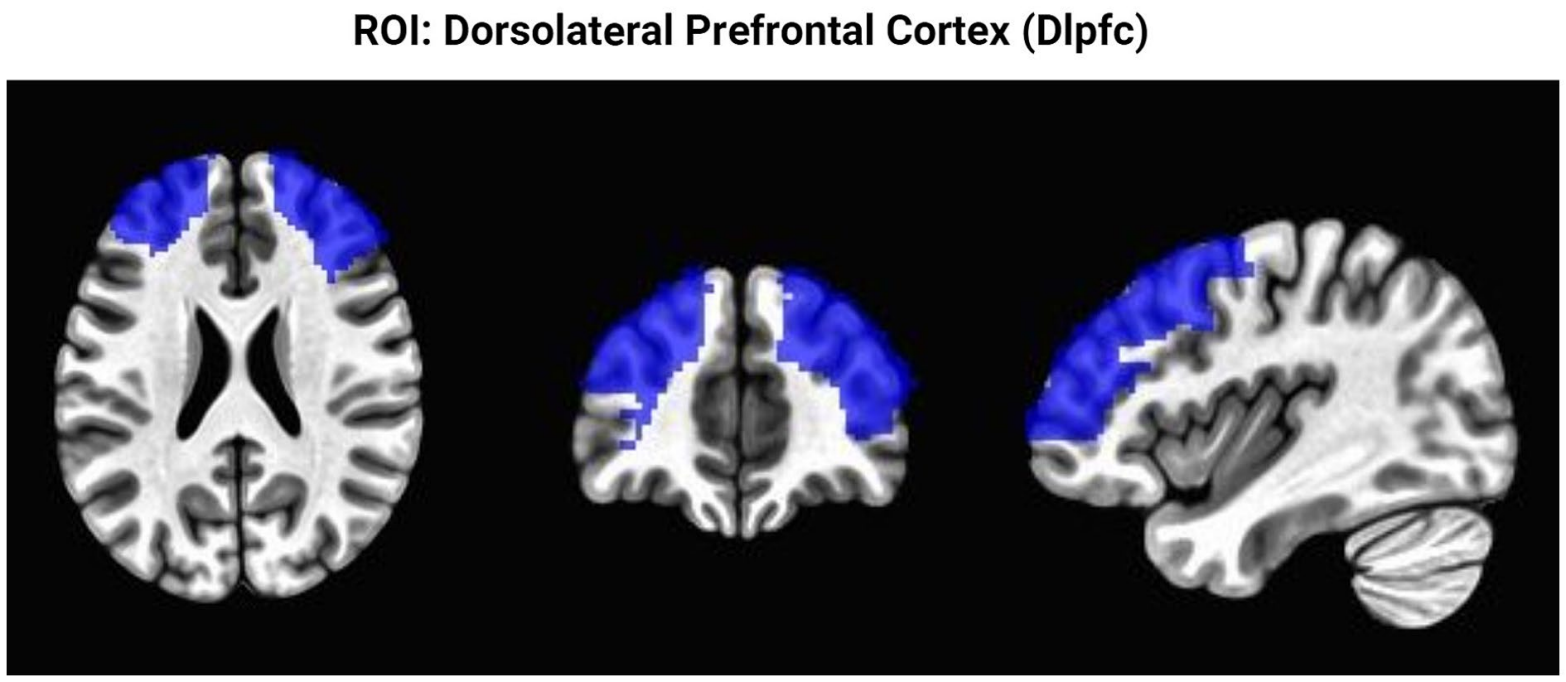
Region of interest analysis. This figure highlights the brain region restricted to perform the ROI analysis. Presented from left to right as axial, coronal, sagittal views.

To test whether practicing SR related to smoking led to brain activation changes in response to food cues, the fMRI percentage signal change for food cues compared to fixation during the ROC task was computed for each participant and entered into the group analysis. The fMRI data were analyzed in the Analysis of Functional Neuroimages program (AFNI) (Cox, 1996), using a linear mixed-effects modeling for group analysis approach (3dLME) with a 2 × 2 within-participant design (Instruction: Positive, Negative; Timepoint: Pre, Post) and one between-group factor (Group: Practice, No Practice). The 3dLME approach allows for the inclusion of data from participants who may be missing data from one time point (Chen et al., 2013). The analyses and corrections for multiple comparisons were restricted to lateral prefrontal cortex regions including the middle frontal gyrus and superior frontal gyrus using AFNI’s whereami to define an anatomical region of interest (ROI; see Figure 3) to determine changes in brain activation within brain regions that manage SR processes. Corrections for multiple comparisons were performed with AFNI’s 3dClustSim. Planned clusters size corrections for multiple comparisons within the prefrontal ROI were set at a conservative voxelwise *p* < 0.001, α < 0.05, and minimum cluster size of 125 mm^3^. However, voxelwise no clusters demonstrating group differences or interactions with group passed this conservative voxelwise threshold. The reported results are corrected for multiple comparisons within the prefrontal cortex mask at a voxelwise *p* < 0.01, α < 0.05 and minimum cluster size 407 mm^3^. Percent signal change data for food compared to fixation were extracted from functionally defined ROIs that showed a Group × Time interaction. A Pearson correlation was performed in SPSS to examine a correlation between adherence and brain activation changes pre- to post- practice delaying the first cigarette. An exploratory analysis was conducted with an ANOVA using IBM SPSS Statistics (Version 26) software to examine changes in self-reported ratings of desire to consume the food images collected during the fMRI. The hypotheses and analytical plan were discussed before performing the secondary analysis of this data.

## 4 Results

### 4.1 Brain activation during food cue-reactivity

**Figure 4:**
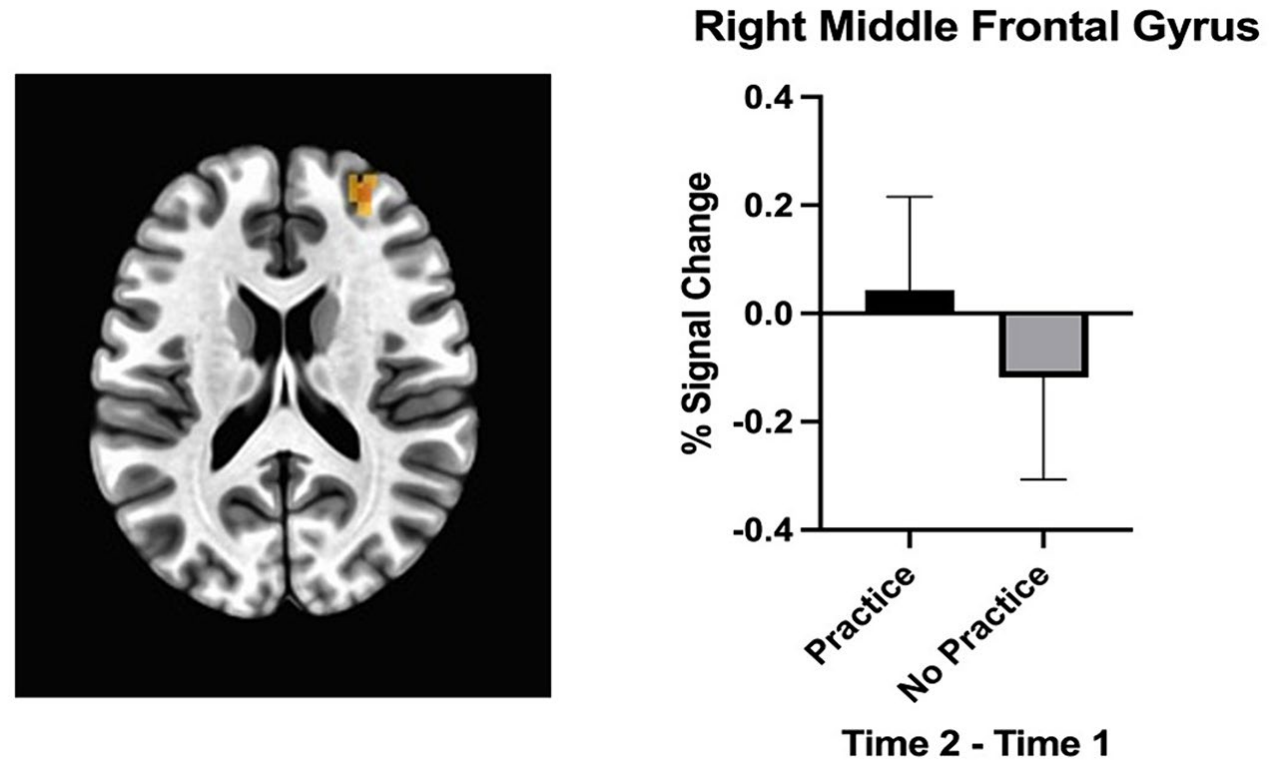
Group × Time interaction. The dlPFC showed a greater change in brain activation for the group that practiced delaying their first cigarette of the day compared to the group smoking as usual. This ROI analysis was performed using AFNI.

The primary analysis examined the effect of practicing SR (i.e., delaying the first cigarette of the day) on brain activation in the dlPFC while participants viewed food cues. A significant interaction of *Group x Time* in the right superior frontal gyrus (SFG) showed greater activation change in the Practice group (delay first cigarette of the day) compared to Control group (smoke as usual; Figure 4). In addition, main effects of *Time* were found in the left SFG and the right SFG near the supplementary motor area (SMA). Left SFG regions showed decreased activation during the follow-up compared to baseline scan, and the right SMA showed greater activation during the follow-up scan. A main effect of *Instruction* was found in the left SFG and showed greater activation when thinking about “positive” compared to “negative” consequences of consuming the item. Table 1 lists all the activation coordinate and cluster information. No significant main effects of *Group* or *Group × Instruction × Time* interaction or *Instruction × Time* interaction were found.

**Table 1:** Brain regions activated during ROI analysis for Group × Time, instruction, and time.

**Regions within cluster**	**L/R**	**mm**^**3**^	***x***	***y***	***z***	**BA**
***Interaction of Group x Time***						
Superior/Middle frontal gyrus	R	578	27	53	18	10
***Main effects of instruction***						
Superior frontal gyrus	L	2,044	−23	33	35	9
***Main effects of time***						
Middle frontal gyrus	L	484	−25	51	13	10
Superior frontal gyrus	L	468	−13	38	53	8
Superior frontal gyrus	L	437	−13	51	40	9
Superior frontal gyrus	R	406	17	3	63	6

Indicating the right or left hemisphere of the brain (L/R). Volume per cluster, Montreal Neurological Institute coordinates (MNI) and Brodmann areas (BA).

### 4.2 Adherence

Within the Practice group (*n* = 24), no significant correlation was found between adherence to delaying the first cigarette of the day and brain activation in the right SFG region that showed a *Time × Group* interaction (*r* = −0.012, *p >* 0.95).

### 4.3 Behavioral analysis

An exploratory analysis examined self-reported ratings from the ROC task. A mixed ANOVA showed a significant main effect for *Instruction* [*F*(1,54) = 95.56, *p <* 0.001, $\eta p^{2}$ = 0.64]. Moreover, self-reported craving ratings for food cues were lower during the “Negative” instruction than for “Positive” instruction (see Table 2). No significant main effects were found for *Time* or *Group* and no significant interactions were found for *Time × Group, Instruction × Group, Time × Instruction or Time × Instruction × Group*.

**Table 2:** Self-reported ratings for the ROC task means.

	**Practice group**	**No practice group**
Baseline positive instruction	3.8 (SD = 0.60)	3.8 (SD = 0.86)
Follow-up positive instruction	3.9 (SD = 0.62)	3.8 (SD = 0.67)
Baseline negative instruction	2.4 (SD = 0.83)	2.5 (SD = 0.86)
Follow-up negative instruction	2.3 (SD = 0.68)	2.5 (SD = 0.93)

## 5 Discussion

Practicing SR by delaying the first cigarette of the day was associated with increased brain activation in the dlPFC, specifically the right MFG region, when viewing food cues. These findings support the hypothesis that SR related to one type of appetitive cue (i.e., smoking) may be generalizable to other appetitive cues (i.e., food). No significant correlation was found between adherence to delaying the first cigarette and change in brain activation.

### 5.1 Self-regulation practice

The MFG is a part of the dlPFC associated with general cognitive processes including memory, emotion regulation, language, attention, cognitive control, decision-making, learning, and more. Specifically, the role of the left MFG has been associated with strategic planning, attentional demand, working memory, inhibition, and emotional processing of verbal stimuli (Boghi et al., 2006; Geng et al., 2006; McNab et al., 2008; Schlochtermeier et al., 2013; Chen et al., 2017; Quinn et al., 2017). Similarly, the right MFG involves attentional control, decision-making, memory, and cognitive control (Wang et al., 2010; Ross et al., 2011; St-Laurent et al., 2011; Robertson et al., 2015). This region is also part of the Ventral Attentional Network (VAN); these neural systems modulate attentional control (Corbetta and Shulman, 2002).

The results from the *Group × Time* interaction suggest differences in brain activation in SR areas between the practice group and no practice group across time points. These findings align with the hypothesis that practicing SR to non-food cues would strengthen SR mechanisms to food cues, such as self-monitoring behaviors like paying attention to their craving levels and environmental cues.

Our results are consistent with models of SR as a generalized process rather than a domain-specific process, such as the Dual systems and the Dynamic models, rather than a domain-specific process like Ego depletion. People who were asked to regulate smoking behavior showed greater brain activation in areas important for SR in response to food cues when it was not the primary goal.

### 5.2 Positive vs. negative instruction

The Regulation of Craving task (ROC) is built on the principles of emotion regulation fMRI paradigms to assess SR brain activation in the context of appetitive cues (Kober et al., 2010). The original task included time expectation for when the food item is consumed, such as “now” or “later.” In previous studies the ROC showed increased brain activation in areas involved in cognitive control and negative emotion regulation like the dlPFC, dorsomedial prefrontal cortex, and ventral lateral prefrontal cortex (Kober et al., 2010). Furthermore, when looking at *Cue × Instruction* interactions, cravings for “Food-Now” were lower than for “Food-Later” and for smoking cues. The version of the Regulation of Craving task used in the present study used “positive” and “negative” instructions instead of “now” and “later.” During the task participants were instructed to think about the “positive” or “negative” consequences of consuming the item presented. This comes from cognitive behavioral approaches that suggest one way to regulate craving is to focus on the negative long-term consequences of consuming an item. Thus, the expectation is that using positive short-term consequences will increase cravings (Sun and Kober, 2020).

The present study found greater dlPFC activation when participants thought about “positive” consequences compared to “negative” consequences which was the opposite of what was expected if “negative” consequences are thought to be associated with increased regulation. Besides noting variations in design and analysis, differences between Kober et al.’s (2010) findings and the current study suggest that excluding the “time” factor (i.e., Now, Later) could account for the discrepancies in brain activation of SR areas (e.g., dlPFC) in the current study compared to prior studies using the ROC task. This highlights the importance of precise task instructions and suggests that some participants may regulate their responses when thinking about “positive” consequences to down-regulate reward-related “positive” associations with the food. The behavioral measures showed greater craving for food items when thinking about “positive” consequences and less craving when thinking about “negative” consequences of consuming the item, thus following the same pattern as the dlPFC activation. The higher activation during “positive” instructions could be related to framing effects. For example, Yokum and Stice (2013) compared differences in craving regulation by asking people to use different cognitive reappraisal techniques. When they asked people to consider the “*benefits of not eating*” compared to “*cost of eating*” or “*suppress craving*,” they found higher levels of brain activation in areas associated with regulation related brain activation [i.e., ventral lateral prefrontal cortex (vlPFC), MFG, middle SFG]. Thus, the effects of the task-framing might be associated with the application of different cognitive reappraisal strategies. In the current study, participants reported thinking about food attributes such as texture, taste, smell, and how the foods made them feel during “positive” and “negative” trials and reported thinking about body image, dietary impact, and cost during “negative” trials suggesting high variability in approach to the instructions.

### 5.3 Adherence

Lastly, there was no evidence to support the influence of practice amount of cognitive control techniques (delay smoking) on brain activation in SR areas such as the dlPFC when attempting to regulate craving for food cues. Although the practice group showed higher brain activation in SR areas across time when attempting to regulate cravings towards food images, we do not have enough evidence to attribute these changes to a specific amount of SR practice.

## 6 Limitations and strengths

Limitations of the study included the focus on only individuals who smoke and the implementation of the “positive” and “negative” instruction variation. There may be differences in reward processing between people who do and do not smoke given that nicotine effects may alter reward related food cue-reactivity. Further, as described earlier, the use of “positive” and “negative” as opposed to “now” and “later” may not be as sensitive to detecting brain activation in SR areas as the original task by Kober et al. (2010). The subjective categories “positive” and “negative” may be too broad. Further investigation on how SR occurs when using positive framing based on the categories participants produced when regulating responses to food cues during the ROC task could provide valuable information in the future. Using a cue-exposure task with the same or other populations could provide useful information and offer versatility that the cue-induced craving task used in this study. This training could be used to explore the effects of cognitive control techniques on populations with ED, considering the nuances between each ED. Adding a consistent eating schedule to reverse the cue that is being trained (e.g., food cognitive control task while smoking as normal). Additionally, keeping the same smoking habits was a requirement to participate but some participants may not have reported drastic changes during the study. All participants received a 10-min cognitive behavioral therapy strategy session which could have diluted effects on SR differences between groups. Finally, this analysis was a secondary analysis, and the original study was not specifically designed to test the generalizability of SR practice from smoking to food cues. Future studies could further address this question by comparing SR practice to smoking and/or food and see if the practice generalizes in both directions as well as explore dosing effects.

## 7 Conclusion

In conclusion, practicing SR by delaying the first cigarette of the day appears to generalize SR brain activation to non-smoking, appetitive food cues. These findings could contribute to theoretical frameworks by supporting a generalizable view of SR, which would be good news for people who wish to regulate cravings for multiple substances simultaneously. Future studies examining different doses of practice are needed to fully understand the relationship between the amount of SR practice and generalizability to non-practiced appetitive cues.

## Data availability statement

The raw data supporting the conclusions of this article will be made available by the authors, without undue reservation.

## Ethics statement

The studies involving humans were approved by University of Kansas Human Subjects Committee (STUDY00004095). The studies were conducted in accordance with the local legislation and institutional requirements. The participants provided their written informed consent to participate in this study. Written informed consent was obtained from the individual(s) for the publication of any potentially identifiable images or data included in this article.

## Author contributions

IT: Writing – original draft, Writing – review & editing, Conceptualization, Resources, Software, Visualization. MP: Writing – original draft, Writing – review & editing, Conceptualization, Supervision, Validation. MB: Data curation, Investigation, Methodology, Project administration, Resources, Software, Writing – original draft, Writing – review & editing. LM: Writing – original draft, Writing – review & editing, Conceptualization, Formal analysis, Investigation, Resources, Software, Supervision, Visualization.

## References

[ref1] Berridge K. C. (2007). The debate over dopamine’s role in reward: The case for incentive salience. Psychopharmacology 191, 391–431. doi: 10.1007/s00213-006-0578-x 17072591

[ref2] Bloom E. L. Farris S. G. DiBello A. M. Abrantes A. M. (2019). Smoking-related weight and appetite concerns and use of electronic cigarettes among daily cigarette smokers. Psychol. Health Med. 24, 221–228. doi: 10.1080/13548506.2018.1537495 30346797 PMC6368221

[ref3] Boghi A. Rasetti R. Avidano F. Manzone C. Orsi L. D'Agata F. P.. (2006). The effect of gender on planning: An fMRI study using the Tower of London task. NeuroImage 33, 999–1010. doi: 10.1016/J.NEUROIMAGE.2006.07.022 17005420

[ref4] Camp D. E. Klesges C. Relyea G. (1993). The relationship between body weight concerns and adolescent smoking. Health Psychol. 12, 24–32. doi: 10.1037//0278-6133.12.1.24 8462495

[ref5] Center for Disease Control and Prevention. (2023). 7 Common withdrawal symptoms and what you can do about them. CDC. Available at: https://www.cdc.gov/tobacco/campaign/tips/quit-smoking/7-common-withdrawal-symptoms/

[ref6] Chen H. Y. Gilmore A. W. Nelson S. M. McDermott K. B. (2017). Are there multiple kinds of episodic memory? An fMRI investigation comparing autobiographical and recognition memory tasks. J. Neurosci. Off. J. Soc. Neurosci. 37, 2764–2775. doi: 10.1523/JNEUROSCI.1534-16.2017 PMC6596641 28179554

[ref7] Chen G. Saad Z. S. Britton J. C. Pine D. S. Cox R. W. (2013). Linear mixed-effects modeling approach to fMRI group analysis. NeuroImage. 73, 176–190. doi: 10.1016/j.neuroimage.2013.01.047 PMC3638840 23376789

[ref8] Cohen J. D. (2017). “Cognitive control” in the Wiley handbook of cognitive control. ed. Egner T.. John Wiley & Sons.

[ref9] Corbetta M. Shulman G. (2002). Control of goal-directed and stimulus-driven attention in the brain. Nat. Rev. Neurosci. 3, 201–215. doi: 10.1038/nrn755 11994752

[ref10] Cosme D. Lopez B. R. (2023). Neural indicators of food cue reactivity, regulation, and valuation and their associations with body composition and daily eating behavior. Soc. Cogn. Affect. Neurosci. 18, 1–12. doi: 10.1093/scan/nsaa155 PMC10074773 33216123

[ref11] Courtney K. E. Schacht J. P. Hutchison K. Roche D. J. Ray L. A. (2016). Neural substrates of cue reactivity: Association with treatment outcomes and relapse. Addict. Biol. 21, 3–22. doi: 10.1111/adb.12314 26435524 PMC4986996

[ref12] Cox W. R. (1996). Software for analysis and visualization of functional magnetic resonance neuroimages. Comput. Biomed. Res. 29, 162–173. doi: 10.1006/cbmr.1996.0014 8812068

[ref13] Drummond D. C. (2000). What does cue-reactivity have to offer clinical research? Addiction 95, 129–144. doi: 10.1046/j.1360-0443.95.8s2.2.x 11002908

[ref14] Eichhammer P. Johann M. Kharraz A. Binder H. Pittrow D. Wodarz N.. (2003). High-frequency repetitive transcranial magnetic stimulation decreases cigarette smoking. J. Clin. Psychiatry 64, 951–953. doi: 10.4088/jcp.v64n0815 12927012

[ref15] Ekhtiari H. Faghiri A. Oghabian M. Paulus M. (2016). Functional neuroimaging for addiction medicine: From mechanisms to practical considerations In eds. Ekhtiari H. Paulus M. Progress in brain research. (Amsterdam: Elsevier), 129–153. doi: 10.1016/bs.pbr.2015.10.001 26822357

[ref16] Fox A. T. Catley D. Richter K. P. Ellerbeck E. F. Brucks M. G. Papa V. B.. (2018). Functional brain activation changes associated with practice in delaying smoking among moderate to heavy smokers: study protocol and rationale of a randomized trial (COPE). Trials 19:623. doi: 10.1186/s13063-018-2984-x 30419931 PMC6233265

[ref17] Fregni F. Orsati F. Pedrosa W. Fecteau S. Tome F. A. Nitsche M. A.. (2008). Transcranial direct current stimulation of the prefrontal cortex modulates the desire for specific foods. Appetite 51, 34–41. doi: 10.1016/j.appet.2007.09.016 18243412 PMC3541023

[ref18] Friedman N. P. Robbins T. W. (2022). The role of prefrontal cortex in cognitive control and executive function. Neuropsychopharmacology 47, 72–89. doi: 10.1038/s41386-021-01132-0 34408280 PMC8617292

[ref19] Geng J. J. Eger E. Ruff C. C. Kristjánsson A. Rotshtein P. Driver J. (2006). On-Line attentional selection from competing stimuli in opposite visual fields: Effects on human visual cortex and control processes. J. Neurophysiol. 96, 2601–2612. doi: 10.1152/jn.01245.2005 16855105

[ref20] Gilbert D. G. Rabinovich N. E. (2003). The Emotional Image Series, Version 1.1 [manual]. Unpublished manual. Department of Psychology, Southern Illinois University: Carbondale, IL.

[ref21] Harris K. K. Zopey M. Friedman T. C. (2016). Metabolic effects of smoking cessation. Nat. Rev. Endocrinol. 12, 299–308. doi: 10.1038/nrendo.2016.32 26939981 PMC5021526

[ref22] Inzlicht M. Werner K. M. Briskin J. L. Roberts B. W. (2021). Integrating models of self-regulation. Annu. Rev. Psychol. 4, 319–345. doi: 10.1146/annurev-psych-061020-105721 33017559

[ref23] Kavanagh D. J. Statham J. Feeney G. F. X. Young R. M. D. May J. Andrade J.. (2013). Measurements of alcohol craving. Addict. Behav. 38, 1572–1584. doi: 10.1016/j.addbeh.2012.08.004 23142210

[ref24] Kober H. Mende-Siedlecki P. Kross E. Weber J. Mischel W. Hart C. L.. (2010). Prefrontal-striatal pathway underlies cognitive regulation of craving. Proc. Natl. Acad. Sci. 107, 14811–14816. doi: 10.1073/pnas.1007779107, PMID: 20679212 PMC2930456

[ref25] Komiyama M. Wada H. Ura S. Yamakage H. Satoh-Asahara N. Shimatsu A.. (2013). Analysis of factors that determine weight gain during smoking cessation therapy. PLoS One 8, 1–6. doi: 10.1371/journal.pone.0072010 PMC3749100 23991026

[ref26] LeCocq M. R. Randall P. A. Besheer J. Chaudhri N. (2020). Considering drug-associated contexts in substance use disorders and treatment development. Neurotherapeutics 17, 43–54. doi: 10.1007/s13311-019-00824-2 31898285 PMC7007469

[ref27] McClernon F. J. Hiott F. B. Huettel S. A. Rose J. E. (2005). Abstinence-induced changes in self-report craving correlate with event-related fMRI responses to smoking cues. Neuropsychopharmacology 30:1940. doi: 10.1038/sj.npp.1300780 15920499 PMC1571136

[ref28] McNab F. Leroux G. Strand F. Thorell L. B. Bergman S. Klingberg T. (2008). Common and unique components of inhibition and working memory: an fMRI, within-subjects investigation. Neuropsychologia 46, 2668–2682. doi: 10.1016/j.neuropsychologia.2008.04.023 18573510

[ref29] Muraven M. Baumeister F. R. (2000). Self-regulation and depletion of limited resources: Does self-control resemble a Muscle. Psychol. Bull. 126, 247–259. doi: 10.1037//0033-2909.126.2.247 10748642

[ref31] Neal A. Ballard T. Vancouver J. (2017). Dynamic self-regulation and multiple-goal pursuit. Annu. Rev. Organ. Psych. Organ. Behav. 4, 401–423. doi: 10.1146/annurev-orgpsych-032516-113156

[ref32] Olney J. J. Warlow M. S. Naffziger E. E. Berridge C. K. (2018). Current perspectives on incentive salience and applications to clinical disorders. Curr. Opin. Behav. Sci. 22, 59–69. doi: 10.1016/j.cobeha.2018.01.007 29503841 PMC5831552

[ref33] Quinn C. Taylor J. Davis M. H. (2017). Learning and retrieving holistic and componential visual-verbal associations in reading and object naming. Neuropsychologia 98, 68–84. doi: 10.1016/j.neuropsychologia.2016.09.025 27720949 PMC5407349

[ref34] Robertson B. Hiebert N. Seergobin K. Owen A. MacDonald P. (2015). Dorsal striatum mediates cognitive control, not cognitive effort per se, in decision-making: An event-related fMRI study. NeuroImage 114, 170–184. doi: 10.1016/j.neuroimage.2015.03.082 25862263

[ref35] Ross R. S. Sherrill K. R. Stern C. E. (2011). The hippocampus is functionally connected to the striatum and orbitofrontal cortex during context dependent decision making. Brain Res. 1423, 53–66. doi: 10.1016/j.brainres.2011.09.038 22000080 PMC3205300

[ref36] Sayette M. A. Tiffany S. T. (2013). Peak provoked craving: An alternative to smoking cue-reactivity. Addiction 108, 1019–1025. doi: 10.1111/j.1360-0443.2012.04013.x 23075138 PMC3549313

[ref37] Schlochtermeier L. Kuchinke L. Pehrs C. Urton K. Kappelhoff H. Jacobs A. (2013). Emotional Picture and Word Processing: An fMRI study on effects of stimulus complexity. PLoS One 8, 1–12. doi: 10.1371/journal.pone.0055619 PMC3569458 23409009

[ref38] St-Laurent M. Abdi H. Burianová H. Grady C. L. (2011). Influence of aging on the neural correlates of autobiographical, episodic, and semantic memory retrieval. J. Cogn. Neurosci. 23, 4150–4163. doi: 10.1162/jocn_a_00079 21671743 PMC3262842

[ref39] Sun W. Kober H. (2020). Regulating food craving: From mechanisms to interventions. Physiol. Behav. 222,1–7. doi: 10.1016/j.physbeh.2020.112878 PMC7321886 32298667

[ref40] Szabo-Reed A. N. Breslin F. J. Lynch A. M. Patrician T. M. Martin L. E. Lepping R. J.. (2015). Brain function predictors and outcome of weight loss and weight loss maintenance. Contemp. Clin. Trials 40, 218–231. doi: 10.1016/j.cct.2014.12.008 25533729 PMC4314339

[ref41] Van den Akker K. Stewart K. Antoniou E. E. Palmberg A. Jansen A. (2014). Food cue reactivity, obesity, and impulsivity: are they associated? Curr. Addict. Rep. 1, 301–308. doi: 10.1007/s40429-014-0038-3

[ref42] Wang L. Liu X. Guise K. Knight R. T. Ghajar J. Fan J. (2010). Effective connectivity of the fronto-parietal network during attentional control. J. Cogn. Neurosci. 22, 543–553. doi: 10.1162/jocn.2009.21210 19301995

[ref43] Yokum S. Stice E. (2013). Cognitive regulation of food craving: Effects of three cognitive reappraisal strategies on neural response to palatable foods. Int. J. Obes. 37, 1565–1570. doi: 10.1038/ijo.2013.39 PMC3709002 23567923

